# Analysis of Fluorescence Decay Kinetics of Indocyanine Green Monomers and Aggregates in Brain Tumor Model In Vivo

**DOI:** 10.3390/nano11123185

**Published:** 2021-11-24

**Authors:** Dina Farrakhova, Igor Romanishkin, Yuliya Maklygina, Lina Bezdetnaya, Victor Loschenov

**Affiliations:** 1 Prokhorov General Physics Institute of the Russian Academy of Science, 119991 Moscow, Russia; igor.romanishkin@nsc.gpi.ru (I.R.); us.samsonova@physics.msu.ru (Y.M.); loschenov@nsc.gpi.ru (V.L.); 2 Centre de Recherche en Automatique de Nancy, CNRS, Université de Lorraine, 54519 Vandoeuvre-lès-Nancy, France; l.bolotine@nancy.unicancer.fr; 3 Institut de Cancérologie de Lorraine, 54519 Vandoeuvre-lès-Nancy, France; 4 Institute of Engineering Physics for Biomedicine, National Research Nuclear University MEPhI, 115409 Moscow, Russia

**Keywords:** fluorescent diagnosis, fluorescent lifetime, near-infrared range, fluorescent dyes, H-aggregates, indocyanine green, brain cancer, glioma C6

## Abstract

Spectroscopic approach with fluorescence time resolution allows one to determine the state of a brain tumor and its microenvironment via changes in the fluorescent dye’s fluorescence lifetime. Indocyanine green (ICG) is an acknowledged infra-red fluorescent dye that self-assembles into stable aggregate forms (ICG NPs). ICG NPs aggregates have a tendency to accumulate in the tumor with a maximum accumulation at 24 h after systemic administration, enabling extended intraoperative diagnostic. Fluorescence lifetime analysis of ICG and ICG NPs demonstrates different values for ICG monomers and H-aggregates, indicating promising suitability for fluorescent diagnostics of brain tumors due to their affinity to tumor cells and stability in biological tissue.

## 1. Introduction

Any surgical intervention in the central nervous system for tumor resection requires high accuracy and selectivity of its effect on tissue. Currently, the main problem is the lack of a rapid, objective, and comprehensive intraoperative assessment of the boundaries of tumor tissue during surgical resection or a laser-induced therapy session [1]. The laser spectroscopic methods provide a unique opportunity to determine noninvasively the most significant parameters characterizing the condition of tissues and the prognosis of its possible evolutionary pathological changes, which may occur in the absence of timely treatment. Optical spectroscopy allows one to obain a wide range of information about physiological and morphological parameters [2,3]. This approach identifies a relationship between data from pathological tissue such as absorption, fluorescence, scattering caused by substances originally inherent to tissues and cells, and externally introduced markers. Laser spectroscopic methods with time resolution allow one to determine the state of a tumor and its microenvironment based on the changes in its photosensitizer (PS) fluorescence lifetime. The difference between the fluorescence lifetime is due to photosensitizer accumulation in cells with different phenotypes [4]. Immunocompetent cells, including macrophages, are a predominant component of brain tumors bioenvironment and allow an enhanced uptake of the photosensitizer molecules compared with cancer cells [5]. The change of fluorescence lifetime of ICG in cancer cells is due to a modification in the refractive index of biological tissues [6]. This approach has the advantage of being less sensitive to changes in the irradiation intensity and the dye concentration than the (relative) fluorescence intensities [7,8,9]. Noninvasive conditions for assessment of brain tumor tissue and surrounding tissues is important for performing a relapse-free operation without reducing the patient’s quality of life. It is important to register small and fast functional changes to assess the relationship between surface metabolic and structural changes that occur during the formation and growth of brain cancer [10].

Exogenous fluorophores with selective accumulation in tumor cells and successive photo-cytotoxic effects can significantly increase the delineation of cancer boundaries and enhance their therapeutic effect. Indocyanine green (ICG) is a well-known infra-red fluorescent dye that is used extensively for vascular system imaging [11], tumor angiogenesis [11,12], and internal organs [13] imaging in vivo. ICG has an absorption in the far-red and near-infrared ranges, where biological components such as water and hemoglobin have low coefficients of absorption and scattering. ICG monomers and H- and J-aggregates have absorption peaks at 780 nm, 715 nm, and 895 nm, respectively, where penetration depth fluctuates from 0.5 cm to 0.8 cm. Unfortunately, ICG is not preferable for tumor diagnosis due to limited accumulation and a high photobleaching effect [14]. There is a lot of research related to ICG embedded in different delivery system such as liposomes [15], polymers [16], lipid nanoparticles [17,18], albumin nanoparticles [19,20], and nanofibers [21], which significantly improve limited accumulation in vivo and photostability. High temperatures enable ICG to form stable H- and J-aggregates [22,23]. At present, there are numerous studies that use ICG colloidal solutions (ICG NPs) for cancer treatment [24,25,26,27]. ICG aggregates have a tendency to accumulate in the tumor, enabling extended intraoperative diagnostic. Moreover, after internalization of the aggregates by cells, it could be disassociated into the monomeric form of ICG [28], which can lead to the destruction of tumor tissue via photodynamic action.

## 2. Materials and Methods

Colloidal solution of ICG NPs and molecular solution of ICG were used as fluorescent dyes. ICG molecular form was heated at 65 °C for 20 h to form ICG NPs according to a previously published method [28]. After forming ICG NPs, the solution was filtered through 0.40 µm syringe filters to remove large aggregates. BALB/c mice with glioma C6 xenograft were used as experimental models. The experimental group consisted of 18 female mice with 20–23 g body weight in the age range of 6–8 weeks. The mice were kept in individually ventilated cages with controlled environmental values.

Animal care guidelines were used under the protocol of European Convention for the Protection of Vertebrate Animals used for Experimental and Other Scientific Purposes (Strasbourg, 18.III.1986). For spectroscopic research, the mice were restrained in tube restrainer configurations. Animals’ supervision was conducted daily and efforts were made to prevent mice from suffering.

Fluorescent dyes of ICG and ICG NPs were administered intravenously into the tail vein at 10 mg/kg dose in 0.2 µL volume for 30 s. The concentrations of ICG and ICG NPs were selected corresponding to the doses used in clinical practice.

The fluorescence spectra of ICG and ICG NPs accumulated in tumor xenograft at different time intervals after fluorescent dyes administration were obtained via LESA-01-Biospec spectroscopic system (Moscow, Russia). The excitation wavelength was 633 nm with 5 mW/cm^2^ power density and a corresponding longpass filter was set at 635 nm for fluorescence registration. The filter used is convenient for fluorescence registration of ICG monomers and H-aggregates. A diffusely reflected signal for fluorescence curves was detected by optical fiber in contact of the distal tip to the tumor area and nearby healthy normal tissue (norma). At least three spectra for each measurement were obtained for statistical analysis. Excretory organs study was carried out after sacrificing the mice according to the protocol.

Fluorescence kinetics of ICG and ICG NPs were recorded by a system based on a Hamamatsu C10627-13 streak-camera (Iwata City, Japan) with a time resolution of 15 ps and laser with 637 nm excitation wavelength and 65 ps pulse. The system works on a time-correlated single photon counting approach. The description of the system is reported in the [29]. The fiber was fixed on a tripod to maintain the working distance between the distal end and the tumor tissue. A 637 nm laser is preferable for excitation of ICG H-aggregates, since it falls on the left shoulder of the absorption peak with the maximum at 715 nm. The absorption peaks corresponding to ICG monomers and H- and J-aggregates are presented in our recent paper [30].

Statistical analysis was conducted with the paired Student’s *t*-test for demonstration of significance of ICG monomers and H-aggregates, accumulated in tumor tissue. The empirical value is t = 4.7, greater than the critical one (t_0.01_ = 2.78, t_0.05_ = 4.6).

## 3. Results

**Figure 1:**
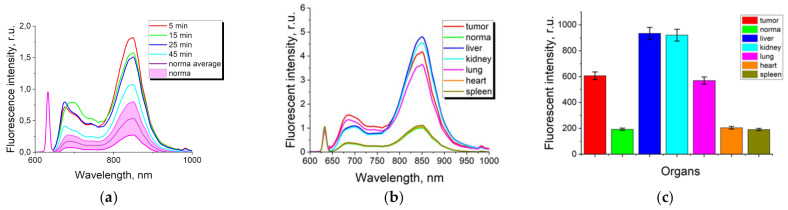
(**a**) Fluorescence spectra of ICG molecular solution in tumor xenografts at different times (λ_ex_ = 633 nm). The data obtained from the study of normal skin samples at all time points fluctuate in the range between two spectral curves (pink color); (**b**) fluorescence spectra of ICG molecular solution in excretory organs 5 min after intravenous administration (λ_ex_ = 633 nm); (**c**) integral dependence of the spectral curve of ICG molecular solution in tumor xenografts 5 min after intravenous administration (λ_ex_ = 633 nm).

The accumulation kinetics of ICG and ICG NPs accumulation in tumor xenografts was assessed according to the fluorescence intensity. Fluorescence spectra were also obtained in the skin, which served as normal tissue sample (Figure 1a). The distribution of the fluorescent dye in excretory organs was obtained at times corresponding to the maximum of its accumulation (Figure 1b,c).

The maximum accumulation of ICG in tumor tissue was observed within the first 5 min after intravenous injection and then the fluorescent dye was rapidly eliminated (Figure 1a). To assess the accumulation of ICG in the excretory organs at the time corresponding to ICG maximum accumulation in the tumor, we registered the fluorescence spectra and the distribution of the fluorescence intensity in the organs and in the skin (taken as normal tissue) (Figure 1b,c). Thus, the absence of significant contrast of ICG is demonstrated since the level of accumulation in the tumor slightly exceeds the level of total accumulation in the skin, while the level of ICG accumulation in the liver and kidneys is the highest, which confirms the presence of rapid elimination processes. The obtained results are consistent with other studies where ICG is used in the clinic as a contrast agent for visualization of the vascular network, which is permissible due to its rapid elimination [11,12,13].

**Figure 2:**
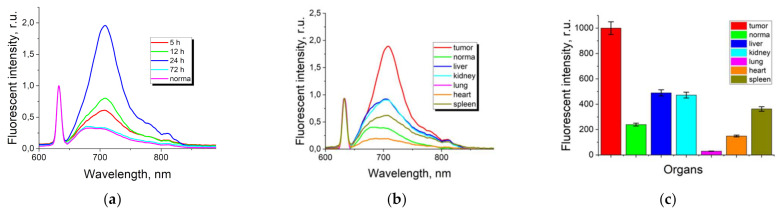
(**a**) Fluorescence spectra of ICG NPs colloidal solution in tumor xenografts at different times (λ_ex_ = 633 nm); (**b**) fluorescence spectra of ICG NPs colloidal solution in excretory organs 24 h after intravenous administration (λ_ex_ = 633 nm); (**c**) integral dependence of the spectral curve of ICG NPs colloidal solution in tumor xenografts under 24 h after intravenous administration (λ_ex_ = 633 nm).

The spectral analysis showed that ICG NPs accumulation in tumor tissue was maximal at 24 h after systemic administration (Figure 2a). Only H-aggregates of ICG were observed at 633 nm excitation after ICG NPs administration corresponding to the fluorescent maximum at 705 nm. The obtained dynamics allow one to conclude that the aggregates enable the fluorescent dye to accumulate and stay in the tumor for a long time (up to 24 h post-administration). It should be noted that the comparison analysis of the fluorescence of ICG and ICG NP at the same time points was not possible. The molecular form of ICG has the rapid clearance by the liver within 1 h after intravenous administration with a rapid successive decline, while the ICG NP fluorescent signal peaks at 5 h after intravenous administration. There is an obvious mismatch between peaks of ICG molecular form and ICG NPs accumulation. The excretory organs analysis demonstrated that H-type aggregates of ICG NP noticeably accumulate in tumor tissue compared with ICG molecular form (Figure 2b,c). As a result of the study, it is worth emphasizing that the aggregated form of ICG NPs is promising from the point of view of its spectral-fluorescent properties, as well as the local contrast accumulation of the nanoform in the tumor.

**Figure 3:**
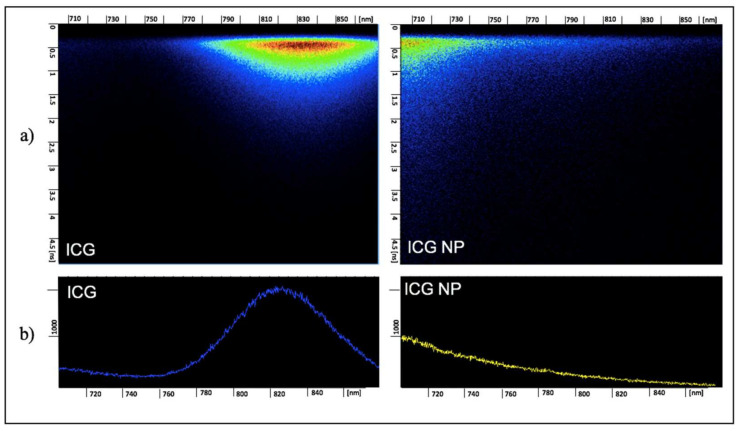
(**a**) Fluorescence spectra of fluorescence of ICG and ICG NPs accumulated in tumor xenograft; (**b**) profiles of fluorescence spectra of ICG and ICG NPs accumulated in tumor xenograft.

The fluorescence lifetimes of ICG and ICG NPs were obtained via the streak camera at times matching the maximal dyes’ accumulation in the tumor (Figure 3).

The spectra of fluorescence lifetime illustrate a fluorescence shoulder of ICG at 700–730 nm, which corresponds to H-aggregates, and an intense fluorescent peak of ICG at 790–860 nm, which corresponds to monomers. For ICG NPs, the fluorescence signal was registered at 700–730 nm, which corresponded to ICG H-aggregates.

The fitting of the obtained fluorescence decay kinetics after the laser pulse excitation was approximated by an exponential function:

(1) $$I\left(t\right)=A_{1}e^{-\frac{x}{t1}}+A_{2}e^{-\frac{x}{t2}}+\cdots$$

where *A*_1_, *A*_2_, … are the amplitude components of the fluorescent lifetime, directly proportional to the contribution of each exponential component; *t*_1_, *t*_2_, … are the corresponding fluorescent lifetime indicators, measured in nanoseconds.

The fluorescence lifetimes of ICG accumulated in tumor xenografts were obtained for H-aggregates and monomers separately, while ICG NPs fluorescence lifetimes were obtained for H-aggregates. Mathematical fitting of fluorescence kinetic spectra demonstrated the availability of two fluorescence lifetimes for each form of ICG. For each sample five spectra were recorded, which were averaged to perform statistical analysis (Table 1). The second column of the Table presents two components of ICG fluorescence lifetimes (ns).

The obtained data show the distinction between fluorescence lifetime values of ICG and different forms of ICG NPs accumulated in tumor tissue. The short component of the fluorescence lifetime of the monomeric form is significantly different from the short component of the H-aggregates. The statistical analysis shows statistically significant differences between the monomers and H-aggregates in the molecular solution. Additionally, the long component of monomers is lower in comparison with the long component of H-aggregates. The obtained data were compared with fluorescence lifetimes in normal tissue after intravenous administration. The fluorescence decay kinetics in tumor tissue are slightly different compared to intact tissue. The fluorescence lifetime of ICG monomeric form in the tumor for both components is slightly higher compared to normal tissue. Meanwhile, H-aggregate values vary within the error margin in tumor and normal tissue.

## 4. Discussion

ICG has significant impact for intraoperative fluorescence navigation in the Near-Infrared-II “window” (1000–1700 nm), especially in brain malignances [10,31,32]. However it has drawbacks like elimination from circulation and low photostability. In addition, once in circulation ICG is rapidly captured by liver, thus limiting its delivery to other sides. This is why the colloidal solution of ICG should be considered due to its stability. The results of the dynamics of the fluorescence signal under 633 nm laser excitation in the tumor after intravenous administration of ICG molecular form demonstrated the maximum fluorescence signal immediately after administration (0–5 min), followed by its uniform elimination within an hour. The studied fluorescent dye ICG in molecular form has promising spectral-fluorescent characteristics necessary for the excitation of deep layers of biological tissues. The fluorescence spectra of ICG NPs in xenografted mice at different time intervals after systemic administration were recorded using λ = 633 nm, which effectively excite H-aggregates for visualization. We demonstrated the kinetics of accumulation of the fluorescent dye ICG NPs, and at the same time, the processes of its interaction with the biological tissue were controlled. The development and testing of ICG NPs colloidal solution is promising in order to increase its specificity to the tumor. ICG molecular form and ICG NPs have various accumulation times in tumor tissue. The fluorescence of ICG molecular form is observed only in the liver within one hour after intravenous administration, while ICG NPs fluorescent signal is detectable in the tumor from 5 h after intravenous administration. The presence of the ICG aggregates is not detectable immediately, but on average, 24 h after intravenous administration demonstrating an intense fluorescent signal in tumor tissue. ICG NPs are expected to be preferentially accumulated by the enhanced permeability and retention effect (EPR) similar to protein-bound ICG molecular form [33]. We hypothesize that aggregates are not internalized by tumor cells but are retained in the extracellular matrix due to surface negative charge of aggregated forms [28]. It was found that ICG NPs tend to be eliminated from the body by the reticuloendothelial system. As we showed in our recent study [30], a high concentration of ICG colloidal solution is observed in the liver and kidneys, indicating the metabolic pathway.

Fluorescence lifetime analysis of ICG and ICG NPs demonstrate different lifetime components for ICG monomers and H-aggregates. The first fluorescence lifetime component of ICG H-aggregate is twice as short as that of the monomer, while the second component of H-type aggregate is longer compared to monomers. According to these data, fluorescent decay kinetics allow to get information on tumor tissue and its bioenvironment. ICG H-aggregates selectively accumulate in tumor tissue, resulting in a clear advantage for long-lasting fluorescent diagnosis. The monomeric form of ICG is mostly used as a contrast agent for visualization of the vascular network, enabling predicting of metastasis ways. While ICG nanoparticles are very promising for fluorescent diagnostics of brain tumors due to their spectroscopic properties and a high selectivity, thus sparing healthy brain tissue.

## 5. Conclusions

To conduct a comparative analysis of ICG in molecular and colloidal solutions, the interstitial distribution of the fluorescent dye was studied in pre-clinical models. The accumulation maximum for ICG and ICG NPs were established in the tumor tissue, in normal tissue, and in the excretory organs. The obtained results showed different accumulation times of the studied fluorescent dyes in the pathological tissue: the molecular form of ICG accumulates within 5 min, while ICG NPs accumulation takes 24 h. It was demonstrated that ICG NPs are promising for fluorescence diagnostics due to their unique optical properties and selective accumulation. The analysis of fluorescence lifetime illustrates the difference between lifetime components allowing to separate ICG monomers and H-aggregates in biological tissue. ICG colloidal solutions have significant prospects for fluorescent diagnostics of brain tumors due to their affinity to tumor cells and stability in biological tissue.

## Figures and Tables

**Table 1:** Fluorescence lifetime of ICG in monomeric and aggregate forms in tumor and healthy tissue.

Sample	Fluorescence Lifetime τ, ns	Amplitude of Fluorescence Lifetime, %
ICG monomers in tumor	0.55 ± 0.09	21%
	0.82 ± 0.03	79%
ICG monomers in norma	0.37 ± 0.09	64%
	0.75 ± 0.05	36%
ICG H-aggregates in tumor	0.28 ± 0.04	83%
	1.10 ± 0.09	17%
ICG H-aggregates in norma	0.27 ± 0.08	82%
	1.21 ± 0.11	18%
ICG NPs H-aggregates in tumor	0.26 ± 0.03	91%
	1.34 ± 0.07	9%
ICG NPs H-aggregates in norma	0.20 ± 0.04	97%
	1.00 ± 0.11	3%

## Data Availability

Not applicable.
